# Cellulose Derived Graphene/Polyaniline Nanocomposite Anode for Energy Generation and Bioremediation of Toxic Metals via Benthic Microbial Fuel Cells

**DOI:** 10.3390/polym13010135

**Published:** 2020-12-30

**Authors:** Asim Ali Yaqoob, Mohamad Nasir Mohamad Ibrahim, Khalid Umar, Showkat Ahmad Bhawani, Anish Khan, Abdullah M Asiri, Mohammad Rizwan Khan, Mohammad Azam, Ahmad Moid AlAmmari

**Affiliations:** 1 Materials Technology Research Group (MaTRec), School of Chemical Sciences, Universiti Sains Malaysia, Penang 11800, Malaysia; asim.yaqoob@student.usm.my; 2 Faculty of Resource Science and Technology, Universiti Malaysia Sarawak (UNIMAS), Kota Samarahan 94300, Malaysia; 3 Center of Excellence for Advanced Materials Research, Chemistry Department, Faculty of Science, King Abdulaziz University, Jeddah 21589, Saudi Arabia; anishkhan97@gmail.com (A.K.); ceamr2@gmail.com (A.M.A.); 4 Chemistry Department, Faculty of Science, King Abdulaziz University, Jeddah 21589, Saudi Arabia; 5 Department of Chemistry, College of Science, King Saud University, Riyadh 11451, Saudi Arabia; mrkhan@ksu.edu.sa (M.R.K.); mhashim@ksu.edu.sa (M.A.); amsa99@gmail.com (A.M.A.)

**Keywords:** benthic microbial fuel cell, energy, synthetic wastewater, graphene oxide, electrode, metals

## Abstract

Benthic microbial fuel cells (BMFCs) are considered to be one of the eco-friendly bioelectrochemical cell approaches nowadays. The utilization of waste materials in BMFCs is to generate energy and concurrently bioremediate the toxic metals from synthetic wastewater, which is an ideal approach. The use of novel electrode material and natural organic waste material as substrates can minimize the present challenges of the BMFCs. The present study is focused on cellulosic derived graphene-polyaniline (GO-PANI) composite anode fabrication in order to improve the electron transfer rate. Several electrochemical and physicochemical techniques are used to characterize the performance of anodes in BMFCs. The maximum current density during polarization behavior was found to be 87.71 mA/m^2^ in the presence of the GO-PANI anode with sweet potato as an organic substrate in BMFCs, while the GO-PANI offered 15.13 mA/m^2^ current density under the close circuit conditions in the presence of 1000 Ω external resistance. The modified graphene anode showed four times higher performance than the unmodified anode. Similarly, the remediation efficiency of GO-PANI was 65.51% for Cd (II) and 60.33% for Pb (II), which is also higher than the unmodified graphene anode. Furthermore, multiple parameters (pH, temperature, organic substrate) were optimized to validate the efficiency of the fabricated anode in different environmental atmospheres via BMFCs. In order to ensure the practice of BMFCs at industrial level, some present challenges and future perspectives are also considered briefly.

## 1. Introduction

Human beings are seriously involved with two main problems: energy inadequacy and less availability of hygienic water due to the wide utilization of non-renewable energy sources and unproductive wastewater technologies. Therefore, it is necessary to adapt an efficient technique which can address both problems simultaneously. Recently, complete protype microbial fuel cells (MFCs) were introduced to generate clean energy and concurrently remove the pollutant ions from water resources [1,2]. MFCs is an approach in which bacterial species serve as catalysts to oxidize the supplied organic substrate to generate the electrons and proton [3,4]. The direct conversion of organic substrates into electrical energy and an efficient operation at a stable environment distinguish MFCs from other current energy devices. The other advantage of MFCs along with energy generation is wastewater treatment application [5,6]. Further, continued efforts have modified MFCs into benthic microbial fuel cells (BMFCs). BMFCs are a type of MFCs in which natural organic substrate is used instead of providing them from outside resources [7]. BMFCs are also known as sediment MFCs (SMFCs) (based on the operational material and parameters), which utilize the naturally present potential difference between oxic seawater and anoxic sediment to generate energy. The microbial community oxidizes the organic substrate in sediment and generates the electrons and protons. Additionally, the supplied organic substrate in BMFCs acts as an inoculum source, proton-exchange membrane (PEM) and nutrient-rich anodic medium. This feature makes it different from other MFCs reactors and types [8]. The abundant accessibility of organic substrates in the sediment becomes a power source for underwater vehicles and independent marine sensors because they are continuously providing the maintenance-free power amount for an extensive time. BMFCs offer a stable voltage trend at an appropriate fast rate of electron transportation. The BMFCs consist of two chambers: the anode chamber is filled with natural organic waste with target pollutant water, while the cathode is filled with aerated tap water. The generated electrons travel to the cathode electrode from the anode electrode by using the outer circuit, while the proton moves directly towards the cathode [9]. Worldwide, energy and water demand are continuously growing, and MFCs and their types (BMFCs) are essential to address the present issues [10]. Therefore, efforts are needed to improve BMFCs’ performance, which can solve some issues such as electron transportation, bacteria growth promotion, and efficient organic substrate at a low cost. The utilization of natural organic substrate in BMFCs could reduce the cost because there are many waste-derived organic substrates freely available. Similarly, a cost-effective highly conductive novel anode electrode is needed to improve the electron transportation and bacteria growth. The anode electrode of BMFCs has direct contact with the bacterial biofilm (biofilm is a layer of bacteria species on the surface of an anode), which generates and transfers the electrons more effectively [11]. Among all factors which carry out a direct effect on bacterial growth, the anode electrode is one of them which can offer a stable environment without any toxicity effects. Carbon-based materials have received great attention in electrochemistry due to their moderate conductivity and surface area [12]. Additionally, in terms of conductivity the metal-based electrode has showed higher conductivity than the carbon-based electrode, but due to the corrosion issue in metal-based electrodes, the long-term stability of the system was decreased. Corroded metal electrodes also generate adverse effects against microorganism growth and their activities [13]. Therefore, carbon-based material dragged the interest to serve as an electrode in BMFCs. Several conventional carbon-based anodes have been used in BMFCs such as carbon rods, veils, foam, sheets, etc., but their energy performance was not adequate [14]. The carbon black and carbon nanotubes (CNTs) showed quite high conductivity compared to conventional carbon, but the bacterial cellular toxicity of these materials makes them unsuitable for BMFCs operation. The bacterial cellular toxicity and susceptibility towards oxidation dragged the interest towards the utilization of graphene derivatives [15]. Graphene derivatives are sp^2^ hybridized carbon atoms which are organized in a honeycomb frame. This carbon class showed high thermal, mechanical, chemical, and moderate electrical conductivity, even more superior than CNTs or other materials. The graphene derivatives also showed biocompatibility towards the bacterial growth and their respiration. The commercial graphene derivatives used to fabricate the anode are very expensive, which has greatly disallowed MFCs’ suitability at commercial scale. Therefore, the synthesis of graphene oxide (GO) from waste material by using the hummers method is a great idea to reduce the cost of GO synthesis. The self-fabricated GO anode showed high energy performance, biocompatibility, and lower cost than other commercial anodes [16].

Furthermore, the GO structure has several oxygen-based functional groups which are present on basal planes and the edges of GO sheets. The electrical conductivity is low through the destructed conjugated sp^2^ hybridized system by the hydrophilic functional group. The electrical conductivity of GO urges the modification of material with conductive polymer which can significantly improve the electrical conductivity and will be suitable as an anode in BMFCs [17,18]. Conductive polymers such as poly(3-hydroxybutyrate-co-3-hydroxyvalerate), poly (aniline-co-o aminophenol), polyaniline (PANI), and polypyrrole were used as anodes to enhance the electronic conduction properties [14,19].

Moreover, in a previous study, Thambidurai et al. [19] stated that the utilization of PANI in energy generation application is preferable compared to other conducting polymers due to its low cost, excellent doping properties, good stability, conductivity, and easy synthesis. Yong et al. [20] have reported PANI-graphene foam composite anode fabrication and achieved 768 mW/m^2^ energy efficiency in MFCs, which is around four times higher than bare graphene foam. The PANI increased the electron transfer rate which led to high energy output. Similarly, Zhao et al. [21] reported the graphene ribbons/PANI based anode catalyst in MFCs. The obtained results showed 6-fold higher energy output than a bare electrode. Therefore, the PANI modification could bring a breakthrough in MFCs.

Unfortunately, there are few studies available on GO-PANI as an anode for BMFCs with natural organic waste. The present article is focused on the synthesis of GO material from cellulose waste, which is very easily available in the ASEAN (Thailand, Malaysia, Indonesia, Cambodia, Vietnam) territory [22]. The fabricated anode for BMFCs produced from synthesized GO with modification by PANI is briefly discussed and well supported by electrochemical measurements. Furthermore, to evaluate the wastewater treatment efficiency of BMFCs by using the GO-PANI anode, a study was performed on Pb (II) and Cd (II) as pollutants of wastewater in the present work. Different parameters (pH, temperature, organic substrate) were also optimized by using the self-fabricated anode to validate the performance at various environmental conditions. In present BMFCs operation, local sweet potato waste was used as an organic substrate. According to our knowledge, there is no work reported on sweet potato waste as an organic substate in BMFCs. Additionally, the present article is limited to metal ions and operation at laboratory scale.

## 2. Experimental Details

### 2.1. Chemical and Materials

The materials were: sweet potato waste material (received from local market of Pulau Pinang Malaysia), cellulose waste material (gift received from materials technology research group (MaTRec), School of Chemical Sciences, Universiti Sains Malaysia, 11800 Minden, Penang, Malaysia), aniline (Merck, 99.5%), *dichloromethane* (90% QRec), potassium permanganate (Sigma-Aldrich), hydrogen peroxide (30–32%, QRec), hydrochloric acid (99%, Qrec, AR grade), sulphuric acid (95–97% QRec), ethanol (approx. 95%, QRec), sodium nitrate (Sigma-Aldrich), filter paper (Adventic 70 mm), polysulfones (PSF) (M_w_~35,000, Sigma-Aldrich), phosphate buffer (pH 7, Qrec, AR grade), ammonium persulfate (APS) (Sigma-Aldrich, >98%), cadmium nitrate tetrahydrate (R&M Chemicals), and lead nitrate (R&M Chemicals). Deionized (DI) water was used for washing purposes.

### 2.2. Preparation of GO

The preparation of GO by using waste material was carried out by following the hummer method [23]. The GO preparation by hummer method is quite easy and inexpensive compared to other methods. The GO product obtained by this process is more organized in structure, no toxic gases are produced during the reaction, and the degree of reduction provides an equivalent level of conductivity when compared to other methods [24]. The received cellulose waste material was treated with a furnace oven at 1000 °C with argon gas flow, and the heating rate was 20 °C/min. The cellulose waste material was placed in a furnace for 3 h to obtain the carbonized carbon powder. After carbonization, the obtained carbon flakes were ground to obtain the carbon powder, which was further oxidized to prepare the GO. In the process of preparation, firstly 5 g of carbonized carbon powder and sodium nitrate (6 g) were magnetically stirred in sulphuric acid (150 °C) for 1 h at 0–5 °C temperature. Later, potassium permanganate (15 g) was added in sulphuric acid by keeping the temperature less than 5 °C for 3 h. The temperature of the reaction was maintained by using an ice bath. The complete oxidation (showing violet brown color) was achieved by placing the reaction mixture on magnetic stirring without an ice bath for 24 h. After that, 150 mL distilled water was added dropwise into the reaction mixture on constant stirring by keeping the temperature at 90 °C until the dark brown color appeared. After this indication, the reaction mixture was cooled down to room temperature and 150 mL water and 30 mL hydrogen peroxide were added to reduce the effect of potassium permanganate. Finally, the product was washed several times by using the distilled water and heated at 40 °C in a simple oven for 24 h to obtain the powdered form of GO, and it was later stored in air-tight teflon vials at 30 °C in a simple oven for 24 h. After that, the synthesized materials were used for GO-PANI nanocomposite preparation and the fabrication of the GO anode. The hummer method was also previously followed by Shahriary et al. [24].

### 2.3. Preparation of GO-PANI Nanocomposite

**Scheme 1:**
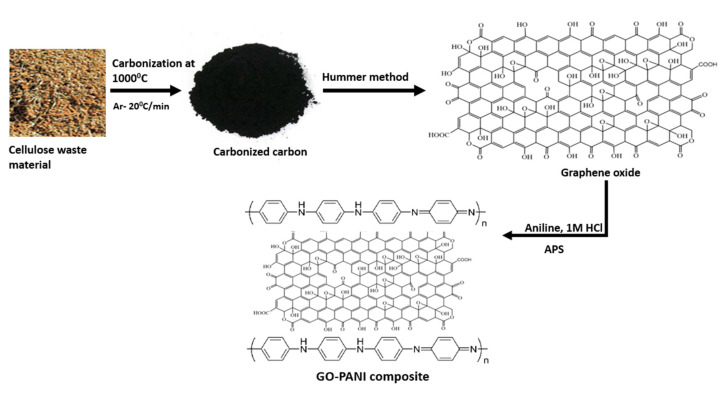
Conversion of biomass to graphene oxide (GO) which leads to formation of GO-polyaniline (GO-PANI) nanocomposite.

A nanocomposite of GO-PANI was prepared by mixing an equimolar of APS and aniline with 100 mL GO solution in order to obtain the homogeneous solution. The aniline was dispersed in 1 M hydrochloric acid. The reaction solution was sonicated for 60 min and then placed in an oven for one day at 25 °C. The obtained solid product of GO-PANI nanocomposite was washed several times by using the distilled water to remove the impurities. Previously, Zheng et al. [25] have also explained GO-PANI nanocomposite preparation by using a similar trend. The biomass conversion to GO-PANI nanocomposite is shown in Scheme 1.

### 2.4. Prepared Material Characterization

The physiochemical and morphological properties of the prepared material are studied by using various techniques. Fourier Transform Infrared (FT-IR; Perkin Elmer model System 2000; Norwalk, CT, USA), thermal gravimetric analysis (TGA-Perkin Elmer Thermal Analyser, model TGA/SDTA 851; Akron, OH, USA), X-ray diffraction (XRD; Philips PW 1710 X-ray diffractometer; New York, NY, USA), UV-spectroscopy (UV-2600i Shimadzu UV-VIS; Kyoto, Japan), scanning electron microscope (SEM-Quanta FEG 650,Fei; Columbia, MO, USA), transmission electron microscope (TEM; Model Zeiss Libra 120; Jena, Germany), and atomic force microscopy ((AFM) SII Sciko Instrument INC SPI 3800N Probe station; New York, NY, USA) are used to assess the material properties.

### 2.5. Fabrication of Anode

The obtained GO and GO-PANI nanocomposite powder were used to prepare the anode for BMFCs. In the present study, the two types of anodes were fabricated to compare their performance. Firstly, the dried fine 5 g GO powder was mixed with PSF (2 g solution) to form a paste which was further coated around the surface of a graphite rod (2 mm). The graphite rod was used as a current collector. The coated GO material on the graphite rod was allowed to heat at 50 °C for 12 h to obtain the solid cylindrical rod-shaped electrode. Similarly, 5 g of GO-PANI nanocomposite was used with 2 g PSF to produce a paste and then coated on a graphite rod to prepare a GO-PANI anode. The surface material of each electrode is responsible for conductivity or other functions, and the graphite rod just served as a current collector [24]. The composition ratio and size were the same in both cases. The measured size (l × w) of each electrode was 7.5 × 1.2 cm with 0.0071 m^2^. The solid cylindrical rod-based design for electrode fabrication offered more advantages than a solid slab design such as flexibility, surface area, and mechanical strength, as explained by Ferdman et al. [26].

### 2.6. BMFCs Inoculation Source, Setup, and Operational Parameters

#### 2.6.1. Inoculation Source Preparation

The inoculation source was synthetic wastewater and sweet potato waste material. The sweet potato waste material was collected from a local market, washed gently and cut into pieces. The sweet potato pieces were used directly in an anode chamber instead of crushed sweet potato waste to serve as an organic substrate for bacterial respiration. On the other hand, the wastewater was collected from a local Humna Geulgor pond which was treated with 100 ppm cadmium and lead ions. The treated wastewater was labelled as synthetic wastewater in the present work. The physiochemical characteristics of the wastewater and synthetic wastewater were also analyzed, which is shown in Table 1. The pH meter (EUTECH instrument-700; New York, NY, USA), electrical meter (ECM) (Alpha-800 conductivity meter; Vernon Hills, IL, USA), and thermometer (GH, ZEAL LTD; London, UK) were used to analyze the pH, conductivity, and temperature.

#### 2.6.2. BMFCs Setup and Operation Parameters

**Figure 1:**
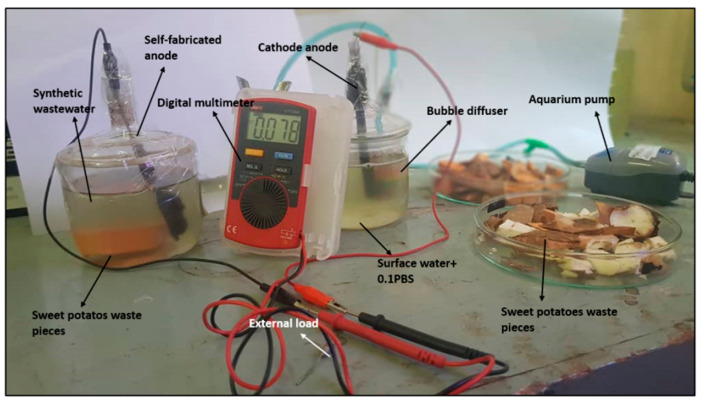
Operational mode of benthic microbial fuel cells (BMFCs) setup used in present study.

The double chamber BMFCs were used in this study. Each chamber has dimensions of 10 × 9.5 cm length and diameter. The total volume space of each chamber was around 1000 mL, but the used working volume was 500 mL. The BMFCs anode chamber was filled with 500 mL synthetic wastewater and 100 g sweet potato waste material, while the cathode was filled with tap water and 0.1 M phosphate buffer. The anode inoculation source pH was 6.45 while the cathode pH was 7, which were maintained by using the phosphate buffer. The anode chamber was sealed without any external inlet while external oxygen was supplied in the cathode chamber. The anode was sealed because there was no need to provide any kind of substrate from outside. Further, the prepared anode electrode was used in each reaction, while the graphite rod (FUDA 2B Lead, NY, USA) was used as a cathode electrode with a similar size to the anode electrode. The distance of the anode and cathode electrode was 7 cm. A platinum (Pt) electric wire was used to transfer the electrons from anode to cathode in the presence of 1000 Ω external resistance. Usually, the internal resistance and external resistance should be comparable. In the beginning of the operation, the internal resistance was gradually low due to the development of the biofilm around the anode [27]. Therefore, there is no hard-fast rule to apply a specific external resistance. Generally, the internal resistance of the cell is dependent on the electrode distance (anode to cathode within the cell) and surface area. According to the previous study reported by Igboamalu et al. [28] regarding the effect of external resistance, the 1000 Ω external resistor is preferable for MFCs operation working at room temperature and a pH of 7. Furthermore, to confirm the precise selection of the external resistance, here 1000 Ω was used before the operation for a few days, and we noticed that the closed circuit voltage was further decreased than the open circuit voltage when loaded by external resistance, but later it recovered slowly. Generally, if it is not recovered, a bigger resistance should be used, and if no significant decrease in voltage takes place, then a smaller resistor should be used in the open circuit voltage. Both BMFC experiments (with GO and GO-PANI anode) were operated in a similar way as described above. Both experiments were operated for 40 days at room temperature. The operational design of the present BMFCs setup is shown in Figure 1.

### 2.7. Electrochemical Measurements

The electrochemical measurements support the material to be recognized as an ideal anode for BMFCs. The voltage (V) of each reaction (GO and GO-PANI) was observed by using the digital multimeter (UNI-T, Model UT120A; Dongguan, China). The potential was recorded every day at the same time (one time in 24 h at 12:30pm) to determine the trend of voltage generation. The Ohm law was used to determine the current efficiency such as power density (PD) or current density (CD). The internal resistance was determined by using Equation (1).

(1) $$r=\left(\frac{E-V}{V}\right)R$$

where *r* = internal resistance, *V* = window voltage, *R* = external resistance and *E* = Electromotive force (Emf). The *E* can be recorded through open circuit voltage (OCV). The polarization behavior was studied by succeeding the “*R*_ext_-variation” scheme, which was well-defined by Logan et al. [2]. A variable resistance box was involved to control the external resistance and was varied from 10,000 Ω to 150 Ω. The polarization curve was analyzed after the pseudo-steady state of the reaction. Generally, 30 min variation time was used during the polarization studies. Further, to study the redox reaction status, cyclic voltammetry (CV; Model BAS Epsilon Version 1.4; West Lafayette, IN, USA) was efficiently used. The CV behavior was observed at different time intervals such as the 10th, 30th and 40th day. The CV scan rate was 10 mV/s and the window voltage range was +0.8 V to −0.8 V. The glassy carbon, Pt and Ag/AgCl electrodes were used as working, counter and reference electrode, respectively. Electrochemical impedance spectroscopy (EIS; Gamry Reference 600; Warminster, PA, USA) was used to examine the resistance effect of the anode towards voltage. EIS analysis was carried out on Day 40 and the frequency range was 100 kHz–100 mHz. Each range needs 20 min to complete the analysis with a scan range of 0.5–0.1 Hz.

### 2.8. Remediation Performance and Biofilm Morphology of Prepared Anodes

The remediation of pollutants from wastewater via BMFCs is also a useful application. The prepared anode also increased the remediating efficiency by empowering the bacterial activities [9]. The metal analysis was performed by using the atomic adsorption spectrometer (AAS) (PerkinElmer A Analyst 400; Waltham, MA, USA). According to schedule, after every 10 days a sample of 1 mL was collected from the anode chamber for AAS analysis. The efficiency was calculated by using Equation (2) by interpreting the AAS results.

(2) $$\mathrm{RE}\;\%\;=\frac{Ti-Tf}{Ti}\times 100$$

where RE is remediation efficiency, *Ti* is total initial metal ions concentration, and *Tf* is final metal ions concentration. The remediation efficiency and energy generation were because of the biofilm growth and development. The biofilm growth and its stability were studied by using the SEM technique. The SEM images showed that the self-prepared anode has a stable biofilm. The SEM images confirmed the bacterial growth on the surface of the anode, which described the biocompatibility of the anode towards living microorganisms. The biofilm around the anode is responsible for the bioremediation of the Cd (II) and Pb (II) from synthetic wastewater. The proper mechanism was previously explained in literature [29,30,31,32]. Furthermore, to identify the bacterial species from the electrode (anode), the produced biofilm was scratched (1.00 mm) from the surface of the self-fabricated anode and kept in distilled water on the completion of BMFCs operation. The serial dilution method was used to transfer the colonies on nutrient agar plates. After some time, nutrient agar plates showed different colonies, which were purified and analyzed very carefully to identify the bacterial species. The pure cultured nutrient agar plates were kept in a fridge until the measurement was carried out. The microbial 16S rRNA genes were achieved by using the polymerase chain reaction (PCR) method. The forward primer (27F) and reverse primer (1492R) were used further to amplify it. A cloning kit (TOPO TA, Invitrogen; Carlsbad, CA, USA) was used to clone the PCR amplified product. After DNA sequencing study, 16S rRNA microbial strains were deposited in GenBank.

### 2.9. Parameter Optimization

In this work, three major parameters were optimized by using self-fabricated anodes (GO and GO-PANI). The optimized parameters were pH, temperature and organic waste because these have a direct effect on the performance of BMFCs. The targeted parameters are very essential to be optimized for GO and GO-PANI anodes because they can directly influence the electron transportation as well as bacteria growth. In optimization, several experimental trails were used to optimize the results in both cases (GO and GO-PANI anodes). In pH, a range from 2–12 (highly acidic to highly alkaline) was used for optimization. The pH was adjusted by using HCL and NaOH. The readings of each range were taken after 10 days (energy and remediation efficiency). During pH study, all other parameters such as room temperature, external load, and electrodes were kept constant. Similarly, in the case of temperature, the experimental trails were carried out in the range of 15–35 °C with pH 7 and 1000 Ω external load. Each temperature range reading and sample was taken after 10 days of time interval for energy output and for remediation analysis. Further, to validate the sweet potato waste as an organic source, another local waste material (tapioca) was used for comparative study. The small pieces of the tapioca waste were used. The readings (energy and remediation efficiency) were observed after 10 days and all other parameters (pH 7, 24 °C, 1000 Ω external load) were kept constant in both cases. During the parameter optimization, we used the same inoculation source for bacterial activities. Additionally, the experiment was performed three times to verify the results.

## 3. Result and Discussion

### 3.1. Material Characterizations

#### 3.1.1. FT-IR Analysis

**Figure 2:**
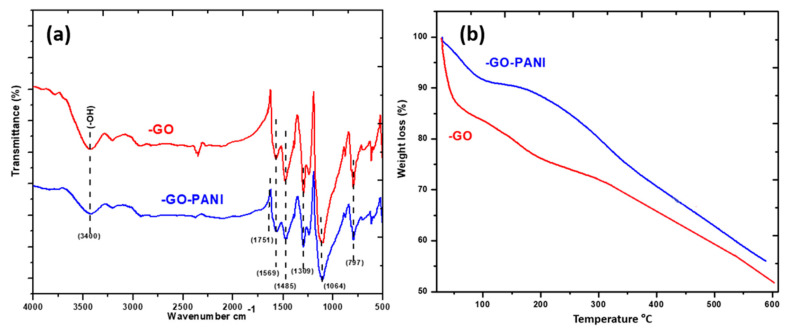
(**a**) Fourier-transform infrared spectroscopy (FT-IR) spectra and (**b**) thermal gravimetric analysis (TGA) spectra of GO and GO-PANI nanocomposite.

The FT-IR spectra of prepared GO and GO-PANI nanocomposite were analyzed within the 500–4000 cm^−1^ region, as shown in Figure 2a. In GO spectra, the -OH group peak was found at 3400 cm^−1^, which confirmed the presence of oxygen in the product. Further, GO spectra demonstrating the presence of C=O (stretching carboxylic group), C=C (stretching vibration), and C-O (stretching vibrations of alkoxy and carboxyl groups) were found at 1751 cm^−1^, 1559 cm^−1^, 1485 cm^−1^ and 1309 cm^−1^ [33,34,35]. On the other hand, a band at 2450 cm^−1^ disappeared in the GO-PANI nanocomposite due to the reshuffling of the structural arrangement after the formation of the composite. The band at 1064 cm^−1^ was attributed to the delocalization of electrons in the C-N vibrational stretching of the quinoid structure. This characteristic indicated the presence of PANI doped with GO structure in the product. Further, the band at 797 cm^−1^ corresponded to the C-H deformation bending of the benzene ring. These types of absorption bands correspond to the PANI [33]. There is no specific different band in GO-PANI, but the peaks were completely red shifted due to the π-π interaction between GO and PANI [36].

#### 3.1.2. TGA Analysis

Figure 2b shows the stability of the prepared material GO and GO-PANI nanocomposite with the help of TGA analysis. The overall observation showed that the GO showed relatively poor stability compared to GO-PANI. The GO-PANI nanocomposite showed 57% stability at 600 °C, while GO showed 50.10%. According to the trend, the decreasing trend of weight is higher in GO than GO-PANI. The GO-PANI showed 57% while GO showed 51% stability at 600 °C. GO showed a major loss around 200 °C, which may be due to the thermal decomposition of the -OH, -COOH, and -CO functional group. Similarly, in GO-PANI a loss was noticed at 100 °C, which is due to the loss of water molecules, while the major loss observed after 300 °C corresponds to the decomposition of PANI. This study showed that the present synthesized material is suitable as an electrode because it will work more effectively up to the maximum temperature of the BMFCs operation [33].

#### 3.1.3. UV-Absorbance Analysis

**Figure 3:**
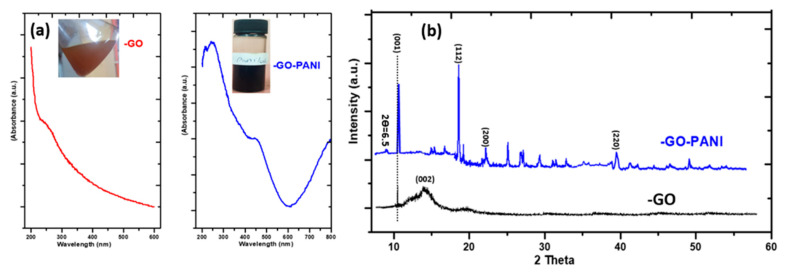
(**a**) UV- absorbance spectra and (**b**) X-ray diffraction (XRD) spectra of prepared GO and GO-PANI nanocomposite.

Figure 3a shows the UV-absorbance spectra of the prepared material, which demonstrates the optical properties. The cellulose-derived GO showed the peak at 240 nm, which corresponds to the π─π* transition of sp^2^ carbons [25]. However, the GO-PANI nanocomposite also showed the narrow peak at 240 nm and the other broad peak appeared at 480 nm, which corresponds to the presence of PANI in the composite. The absorption peak at 480 nm is also attributed to the formation of exciton in quinoid rings, while the other peak at 240 nm refers to the π-π transition in benzene structure. In the GO-PANI nanocomposite, all peaks are red shifted, which shows the coordination of the GO sheet with PANI N-atom [37]. The attachment of PANI with the GO structure improved the electron transfer rate. The electron transportation rate was validated by calculating the band gap. The low band gap exhibited high conductivity as compared to the high band gap. Equation (3) was used to determine the band gap. This equation originates from the Kubelka–Munk theory.

(3) $$Eg=\frac{\mathrm{hc}}{\lambda }$$

where λ is wavelength, c is velocity of light, and h is Planck’s constant. The calculated band gaps of GO and the composite were 5.17 eV and 2.58 eV, respectively. The GO-PANI nanocomposite was referred to as a highly conductive material as compared to unmodified GO.

#### 3.1.4. XRD Analysis

The Figure 3b shows the XRD spectra of the prepared GO and GO-PANI nanocomposite. The GO XRD spectra showed a peak at 10.2°, which is attributed to the effective oxidation of carbonized carbon. The peak at 001 was a clear indication of the synthesis of GO, as already explained in UV. The brownish black end color gives a peak at 10.2°, while another peak at 002 also appeared, which generally corresponded to the graphite oxidation process [33]. The peak shifting from 002 to 001 showed that the GO has a high interlayer space with oxygen functional groups. In another case, the peaks at 15.1°, 19.25°, 25.60°, and 38.0° correspond to the PANI, as previously reported by Yan et al. [38]. The peak at 2Ɵ = 6.5 indicated the presence of a long chain of PANI and with well-ordered structure. The explained 2Ɵ peaks in literature, which are also present in the XRD spectra of the GO-PANI nanocomposite, clearly confirmed the presence of PANI in the composite. The GO-PANI nanocomposite showed a sharp peak at 19.25°, which exhibits an inter-spacing of 3.4A° and indicates that the GO-PANI nanocomposite is more crystalline than GO. This feature showed that the introduction of PANI improved the crystalline property of the material, which might be due to the smooth and slow aniline polymerization process at high temperatures.

#### 3.1.5. SEM and TEM Analysis

**Figure 4:**
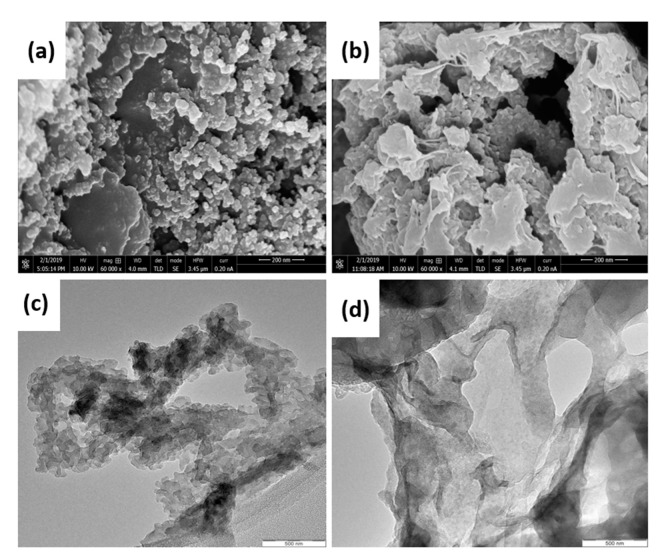
(**a**) Scanning electron microscopy (SEM) image of GO; (**b**) SEM image of GO-PANI nanocomposite; (**c**) transmission electron microscopy (TEM) image of GO; (**d**) TEM image of GO-PANI nanocomposite.

The SEM images were observed to study the prepared GO and GO-PANI nanocomposite morphology. The prepared GO structure showed nano particle shaped morphology as previously reported [39,40,41,42]. The SEM image (Figure 4a) indicates that the size of the particle is in nano range. Similarly, the SEM image (Figure 4b) shows the fibrous morphology of the GO-PANI nanocomposite. The composite image clearly indicates the presence of PANI on the surface of the GO sheet, which appeared like a crystalline rod in the structure. The SEM image shows the successful formation of the GO-PANI nanocomposite. The PANI fibers seem to be randomly distributed between or on the surface of the GO sheet in Figure 4a. However, PANI showed a very well organized and compact structure, as already indicated in XRD spectra. The compact and organized structure corresponds to the close bonding between GO and PANI, which is expected to enhance the electron transportation during BMFCs operation [34].

Further, TEM analysis was carried out to demonstrate the GO sheet structure, which has several scrolls and wrinkles as shown in Figure 4c. The TEM image of GO shows the excellent transparency level, which corresponds to the successful oxidation of carbon. The GO structure showed a thin film structure with a distinctive folding nature because the conjugated GO structure was destroyed during the oxidation. However, Figure 4d shows bit tubular morphology in the composite material, which indicates the presence of PANI. Some flaky structures are also found, which also indicates the successful polymerization of aniline and composite formation with the GO sheet [43]. This nanocomposite formation was successful as the electrode is expected to enhance the electron transportation and collection of electrons.

#### 3.1.6. AFM Analysis

**Figure 5:**
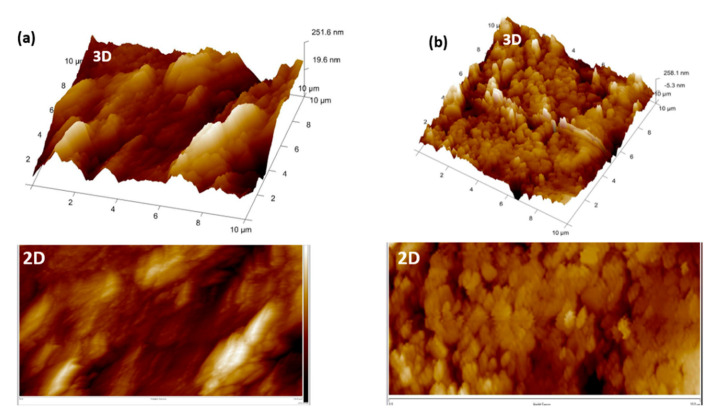
Atomic force microscopy (AFM) images of (**a**) GO (**b**) GO-PANI nanocomposite.

Figure 5 demonstrates the AFM images of GO and the GO-PANI nanocomposite. The three-dimensional (3D) and two dimensional (2D) images exhibit the smooth surface area of the prepared material. Figure 5a exhibits an asymmetrical arrangement of the GO layers, while Figure 5b shows a smooth and fair surface of the material (GO-PANI)**.** The particle arrangement in the nanocomposite was regular and well-arranged, which offered more biocompatibility for bacterial growth in our study. AFM images also confirmed that the synthesized material was well organized with a compact structure and surface. This is a good indication for BMFCs operation to use this material as an anode. Therefore, structural as well as morphological characterizations of the synthesized material reflected a preferable raw stuff as anode electrode formation.

### 3.2. Self-Fabricated Electrode Performance in BMFCs

#### 3.2.1. Voltage Generation Trend and Polarization Behavior

**Figure 6:**
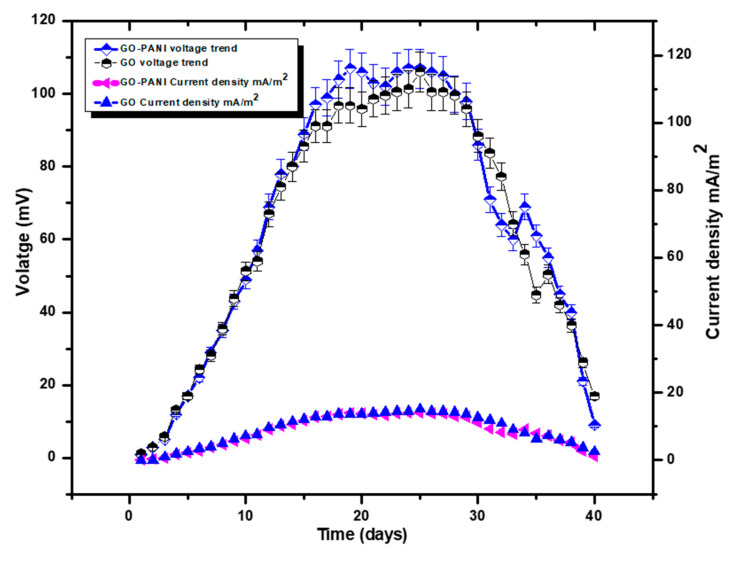
Potential voltage trend of the fabricated anodes in continued operation of BMFCs.

The self-fabricated anodes GO and GO-PANI were used in BMFCs to observe the voltage generation trend. Each experiment was operated for 40 days in the presence of local waste as an organic substrate (sweet potato waste). In the first 5 days of the operation of both the electrodes, a minor difference in voltage generation was observed, but with the passage of time the voltage generation difference increased. For example, on day 5 the GO showed 17 mV and GO-PANI showed 19 mV, which is a minor difference, but on day 25 GO showed 107 mV (0.107 mA) and GO-PANI exhibited 125 mV (0.125 mA). The voltage trend increased gradually in both cases, but the GO-PANI showed quite high voltage generation as compared to unmodified GO. This means that the voltage gradually increased because the supplied organic waste oxidized slowly by bacterial community. The results obtained from the GO-PANI nanocomposite demonstrated that the introduction of PANI increased the electron transportation and it was also biocompatible, which does not show any toxicity towards living microorganisms. The operation of both the electrodes was carried out until the voltage trend deceased. Both electrodes showed a decreasing trend of voltage generation after 25 days, and on day 40 the voltage was very low in both cases. The reason for the gradually increasing trend was due to the supply of organic substrate at one time in the anode chamber, where bacteria rapidly started the oxidization process and simultaneously the electron transportation was high. The novel fabricated anode helped to transfer the electron more rapidly as generated by bacteria during organic substrate oxidization. After a specific point, both electrodes showed less performance due to insufficient supply of organic substrate. This means that the supplied organic substrate was only enough for 25 days to achieve the maximum voltage generation, and after that the low carbohydrate content for the oxidization process decreased the bacterial activity, which led to low generation of electrons. Figure 6 also shows that the current density (CD) gradually increased and then decreased after a specific point, which is an indication of the completion of BMFCs operation. Additionally, the highest recorded power density under the closed-circuit condition was 0.0016 mW/m^2^ with 1000 Ω external resistance.

**Figure 7:**
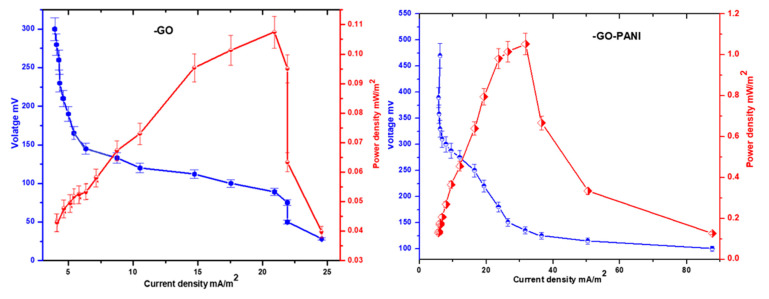
Polarization curves of the fabricated anodes.

Additionally, the polarization behavior was also studied to investigate the relation between current and resistance during the BMFCs operation. A different range of external resistance was used for open circuit voltage measurement. The polarization curve of MFCs contains three different regions, such as activation polarization (the cell potential drop at low power density), ohmic losses (at moderate current density the potential drops slowly), and concentration polarization (at high current density the potential trend drops linearly with current). Further, in the present study, the external resistance was varied from 10 kΩ–150 Ω to plot the polarization curves to find out the appropriate resistance and current relation. The polarization behavior was studied during the operation on day 25 when the potential trend was high in both cases (GO anode and GO-PANI anode). The high external resistance showed the low power generation, but voltage stabilization was rapid while low external resistance showed the high voltage generation, but the voltage was not precisely stable. In GO anode cases, the highest CD 24.56 mA/m^2^ at 150 Ω external resistance was observed and the highest observed power density was 0.107mW/m^2^. The internal resistance of the solution in the GO anode was 2260 Ω. The GO showed an increasing trend of CD from higher to lower external resistance. The high external resistance e.g., 10 kΩ showed 3.94 mA/m^2^, while 9 kΩ showed 4.09 mA/m^2^ and 300Ω showed 21.92 mA/m^2^. Similarly, the GO-PANI anode showed the highest CD (87.71 mA/m^2^) at 150 Ω external resistance with 750 Ω internal resistance effect. The maximum observed power density was 1.05 mW/m^2^ in the case of the GO-PANI anode. The CD of 6.13 mA/m^2^ and 87.71 mA/m^2^ at 10 kΩ and 150 Ω, respectively, were observed, which showed that lower resistance increased the voltage performance (Figure 7). The main limitation of our study was high ohmic losses and the voltage potential trend was dropped linearly. Therefore, the resistance should be lower because it also hinders the electron transportation during transfer from bacteria to anode surface. A consistent voltage trend is required for a stable state. If the environment of the cell is fluctuating, the trend of the current/voltage may be changed. Therefore, to keep the environment stable, fuel cells may take a while to stabilize after the current/voltage variation. Further, the internal resistance is also dependent on anode design (surface, material, nature), electrode conductivity, both electrodes’ spacing distance, electrolyte, and pollutant concentration. Previously, a similar trend of the polarization studies was explained in other studies [44,45]. Further, comparisons between the current density efficiency of anodes in BMFCs and other previously reported anodes are shown in Table 2. In comparison, we found that the fabricated anodes showed less power density efficiency (under close circuit conditions) as compared to other conventional anodes, which might be due to less stability of the organic substrate. The current density of the present work is higher as compared to previously reported work.

#### 3.2.2. Electrochemical Measurements

**Figure 8:**
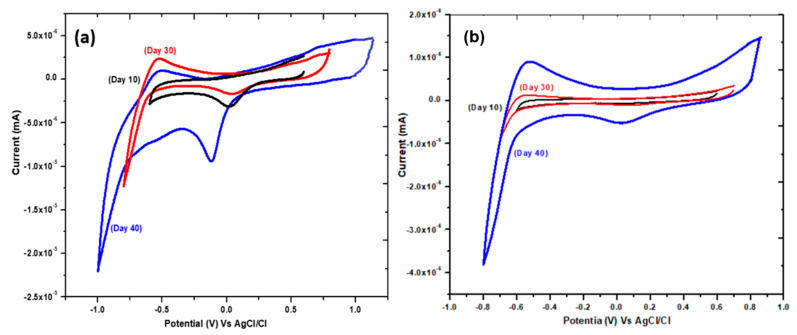
Cyclic voltammetry (CV) study of (**a**) GO (**b**) GO-PANI anodes at different time intervals during BMFCs operation.

The CV analysis was carried out to examine the electron transportation rate. The CV was carried out within +0.8 to −0.8 V potential range with 10 mV/s. The PBS (pH 7) was used as an electrolyte solution in operation. Figure 8a,b demonstrates the CV behavior of both anodes at different time intervals during the BMFCs operation. The CV of GO as shown in Figure 8a depicted both forward scan (F.S.) and reverse scan (R.S.). The F.S. was recorded on day 10 (1.4 × 10^−6^ mA), day 30 (2.9 × 10^−6^ mA), and day 40 (1.1 × 10^−6^ mA), while R.S. was also recorded on day 10 (−1.1 × 10^−6^ mA), day 30 (−1.3 × 10^−5^ mA), and day 40 (−2.3 × 10^−5^ mA). Similarly, the F.S. for the GO-PANI composite was recorded on day 10 (0.3 × 10^−6^ mA), 30 (0.9 × 10^−6^ mA) and 40 (1.3 × 10^−6^ mA), while the R.S. was also recorded on day 10 (0.1 × 10^−6^ mA), 30 (1 × 10^−6^ mA) and 40 (−3.9 × 10^−6^ mA). In GO, the highest F.S. was recorded as 2.9 × 10^−6^ mA and the R.S was −2.3 × 10^−5^ mA, while GO-PANI showed 1.3 × 10^−6^ mA highest F.S. efficiency and −3.9 × 10^−6^ mA as the highest R.S. efficiency. The F.S. or R.S. mean the oxidation and reduction rate at different time intervals of BMFCs operations. The oxidation and reduction peaks are clearly shown in Figure 8a,b. The oxidation peaks of GO and GO-PANI were reported on day 10 (0.6 mA and 0.7 mA), 30 (0.9 mA and 0.8 mA) and 40 (1.1 mA and 0.9 mA). Similarly, the reduction peaks of GO and GO-PANI were also reported on day 10 (−0.60 mA and 0.65 mA), 30 (0.70 mA and 0.79 mA) and 40 (−1.0 mA and 0.81 mA). Day 40 demonstrated the maximum reduction rate, which was attributed to the reverse electron transportation. Day 40 showed the maximum oxidation/reduction due to the external oxygen supply to the cathode, which increased the discharge rate of electrons and also neutralized the electrons before transferring back to the anode [52,53].

**Figure 9:**
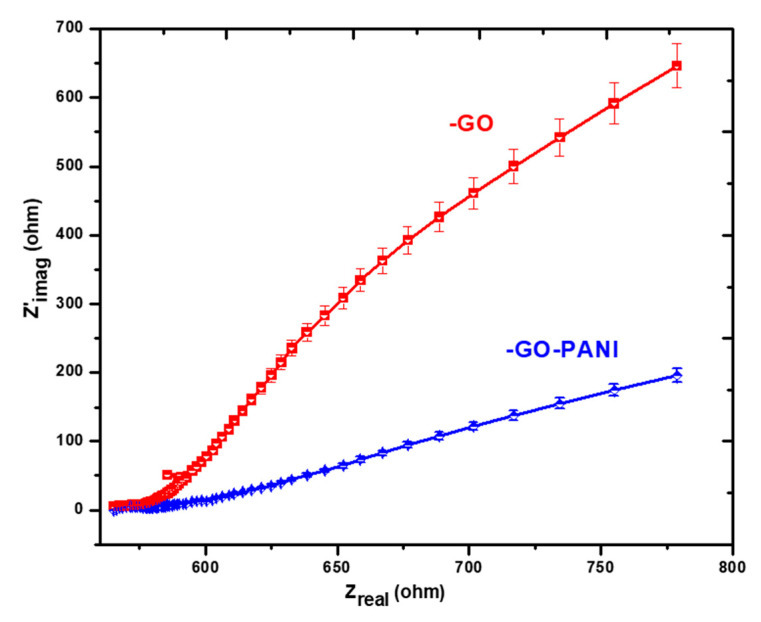
Nyquist-electrochemical impedance spectroscopy (EIS) analysis of the fabricated anode.

Further, the EIS analysis was considered to investigate the charge/transfer resistance activities of the prepared anodes in BMFCs operation. The Nyquist-EIS design was studied in the 100 kHz to 100 mHz frequency range with 1.00 mV AC amplitude. It reduced the biofilm disturbance during EIS analysis and stopped the biofilm detachment effect [54]. Figure 9 shows the observed Nyquist-EIS plot for the GO and GO-PANI anodes, which were affected by kinetic reaction and diffusion with solution resistance (R_s_) and charge-transfer resistance (R_ct_). The EIS spectra usually showed that the straight line with higher Z`_img_ (Ohm) demonstrated low electron transfer, while bent or lower Z`_img_ (Ohm) showed high electron transportation. According to reported results, the GO-PANI showed lower electron resistance in solution than GO. It showed high electron mobility because it showed low (750 Ω) internal resistance in solution. The lower internal resistance referred to the high electron transportation between the electrode and solution. However, the GO showed high internal resistance, which decreased the electronic mobility between the solution and anode. This study concluded that the low internal resistance showed a high rate of kinetic reaction with low charge resistance. This feature demonstrated that the GO-PANI is preferable to enhance the electron transfer rate for high energy performance.

#### 3.2.3. Remediation Performance of Metal Ions via BMFCs by Using Fabricated Anode

The BMFCs were very rarely reported so far in the bioremediation of metal ions from wastewater. The presence of Cd (II) and Pb (II) ions in water resources has caused severe diseases, which are dangerous for human survival [55]. The present study focused on the removal of the most toxic metal ions (Cd and Pb) from synthetic wastewater via BMFCs. The BMFCs were energized by sweet potato waste material. The natural waste as an organic substrate with highly conductive novel anodes was used in BMFCs to convert the metal ions from soluble state to insoluble state. The present study showed that the prepared materials showed good biofilm around the surface because the removal efficiency was excellent, as shown in Table 3. The GO-PANI anode showed high remediation efficiency (65.51%) of Cd (II) as compared to unmodified GO (55.00%). A similar effect was observed in the remediation of Pb (II) since the GO-PANI showed remediation of 60.33% while GO showed 53.50%. In both cases, the GO-PANI showed high remediation performance. This means that the introduction of PANI showed better biocompatibility for the growth of the bacterial community as compared to unmodified GO. Overall, both electrodes showed better biocompatibility for bacterial growth and their respiration process, but the remediation efficiency was quite low as compared to previous studies. After a certain period, the provided organic waste failed to provide enough energy to bacteria for their respiration process. The insufficient supply of organic substrate led the bacterial species to the death phase, which stopped the conversion of toxic metal ions into insoluble state. Singh et al. [56] extensively studied the effects of different organic substrates and concluded that enough supply of organic substrate may lead to poor MFCs efficiency. It can be concluded that the presently used organic substrate failed to supply enough sources for bacterial activities.

#### 3.2.4. Studies of Electrode Biofilm and Bacterial Species Analysis

**Figure 10:**
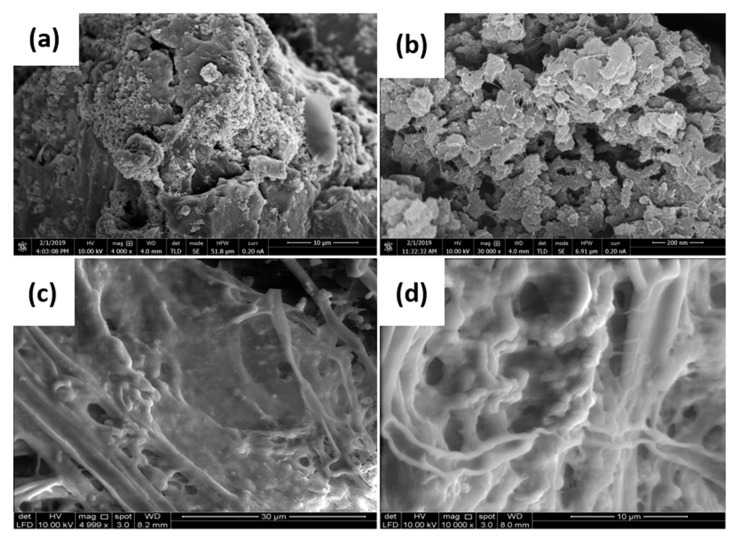
SEM images of fabricated anode biofilms before and after BMFCs operation (**a**) untreated GO anode **(b)** untreated GO-PANI anode (**c**) treated GO anode (**d**) treated GO-PANI anode.

The energy generation and metal ion conversion are both factors that directly depend on the strength and quality of the biofilm. The biofilm is a bacterial layer around the surface of the anode. Biofilm formation does not require any special treatment and it was produced naturally during the BMFCs operation. The biofilm matrix contains 97% water content, 2–5% bacteria cells, 3–6% extracellular polymeric substance (EPS`) and some mineral ions in trace amount [57]. The EPS is one of the factors which hold the bacteria on the surface of the anode for electron generation. The EPS is made up of polysaccharides (40–95%), proteins (1–60%), nucleic acids (10%) and lipids (40%) [58,59]. Furthermore, EPS is a hydrated part of biofilm, which hold the 97% water content via hydrogen bonding. The EPS amount in biofilm significantly enhances the age of biofilm, and when organic substrate supply becomes poor, the EPS will start reduction, which leads to poor efficiency of BMFCs operations. The biofilm should be healthy and efficient to achieve high performance via BMFCs. The prepared anodes GO and GO-PANI were analyzed by using SEM technique to examine the biofilm growth on the surface. As shown in Figure 10a–d, the fabricated anodes were examined before and after the operation, which clearly showed a dense growth of biofilm around the anode surface. The bacterial community successfully produced a biofilm layer around both surfaces, which showed that the fabricated anodes are highly biocompatible for living organisms without any toxic effect. Both electrodes showed mixed culture bacterial species, but SEM images showed some filamentous appendages. The filamentous appendages are conductive pili which help to transfer the electrons from bacterial cell to anode. The electron transfer mechanisms are well explained in our previous study [5]. Therefore, the anode-cell contact is very important for high performance of BMFCs. Further, in the bacteria identification process, it was found that a mixed culture of bacteria was available in the present study. Some of the most dominant species are shown in Table 4, which were observed through the realization of clone library. The presence of mixed culture is a good sign for effective remediation as well as energy generation. Due to the similar incolumn source, both anodes showed almost similar species of bacteria e.g., *Bacillus* species, *Acinetobacter* species, *Proteus* species.

According to Nimje et al. [60] *Bacillus* strains can produce energy, and they recorded a 0.000105 mW/m^2^ power density, which is lower than the recorded results of this study. The present results are higher, which might be due to the utilization of organic substrate or novel anode electrodes. Similarly, Ayangbenro et al. [61] clearly concluded that *Bacillus* species are very effective for the treatment of heavy metals such as Cd and Pb. Similar, several studies showed that the *Acinetobacter* species are responsible for the generation of electricity, as they are mostly found in MFC’s biofilm [3,5]. For example, Fedorovich et al. [62] studied the mixed culture bacteria from MFCs operation and concluded that *Acinetobacter* species are one of the exoelectrogens in their study to achieve a maximal power density of 296 mW/L. Rabaey et al. [63] isolated the *Sphingobacterium* sp. and *Acinetobacter* sp. extensively in their study and found that they are exoelectrogens species with generation of 0.015 ± 0.001 to 0.049 ± 0.025 W m^2^ power density. According to previous studies, the found species (Table 4) can also generate energy as well as remediate the heavy metals effectively [64,65,66,67,68,69].

### 3.3. Multiple Parameter Optimization

The multiple parameter optimization is very important to evaluate the performance of the BMFCs operation, whether the used novel electrode and natural organic waste are preferable or not at different conditions. Each parameter was analyzed by using the self-fabricated anodes with 1000 Ω external load. The inoculated source was synthetic wastewater which contained the Cd (II) and Pb (II) metal ions.

#### 3.3.1. Effect of pH on Energy Generation and Remediation Efficiency of Cd (II) and Pb (II)

**Figure 11:**
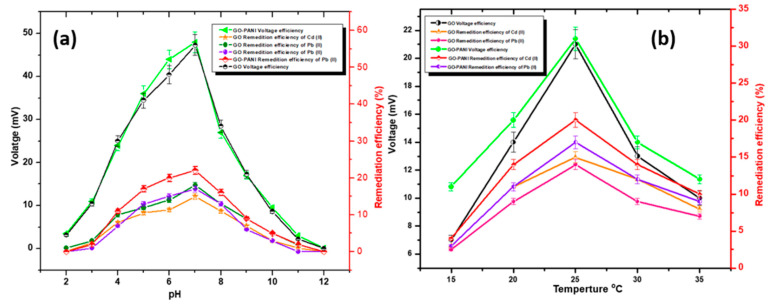
Effect of (**a**) pH and (**b**) temperature by using the self-fabricated anode at different ranges.

The pH carries a direct effect on the energy generation and remediation efficiency of Cd (II) and Pb (II) from synthetic wastewater via BMFCs. Figure 11a shows that energy generation was high at pH 7 rather than highly acidic or basic conditions. Therefore, researchers mostly used buffer solution to maintain the pH of the solution to keep the performance acceptable. According to observations, the pH 2, 3 and 10, 11 and 12 did not provide preferable conditions for the healthy growth of biofilms around the anode surface. For example, at pH 2, voltages of 3.4mV and 4.5 mV were achieved in both GO and GO-PANI, respectively, while pH 12 offered voltages of 0.1 mV and 0.9 mV. The observed voltage was very low which might be considered as zero%. As compared to highly acidic or alkaline conditions, the pH 7 offered voltages of 48 mV (GO) and 56 mV (GO-PANI) after 10-day operation, which are higher than the values found at pH 2,3 or 10,11 or 12. Additionally, one more aspect in the present study is that some energy output was achieved at highly acidic (pH 2) and highly basic (pH 12) conditions. Similarly, pH carries an effect on the remediation efficiency of Cd (II) and Pb (II) from synthetic solution. The neutral pH (7) offered high remediation efficiency e.g., GO showed 15% removal of Cd (II) ions and 17% Pb (II), while GO-PANI showed 18% (Cd) and 22% (Pb) removal efficiency after 10-day operation of BMFCs. In highly acidic medium (pH 2), zero% removal efficiency of Cd (II) and Pb (II) was observed, while pH 12 also showed zero% removal efficiency of both metal ions. The pH disturbed the biofilm, which reduces the bacterial activities that led to poor remediation efficiency as well as poor energy generation [66]. Huang et al. [67] reported the pH effect on the energy performance and removal efficiency of Cd (II) via MFCs. They concluded that the pH 7 can generate significant energy output as compared to pH 2–5 or more than pH 9. They also showed zero% removal efficiency at pH 2 and pH 10 due to the disturbance of biofilm. An acidic or alkaline environment is not feasible for living organisms to survive without any special care. In another study, Yuan et al. [68] also reported the effect of pH on the formation of biofilm which is liable for removal efficiency. They also studied at pH 5 and pH 9, and they observed that the pH 5 is not preferable as compared to pH 9 because they achieved 2.5 times higher energy efficiency at pH 9 as compared to pH 5. In the present study, higher energy and remediation efficiency were observed at pH 4.5 as compared to pH 9, 10, or 11. In the present study, each range of pH reading (voltage and remediation analysis) was taken after 10 days of operation.

#### 3.3.2. Effect of Temperature on Energy Generation and Remediation Efficiency of Cd (II) and Pb (II)

Temperature also showed a significant effect on energy performance via BMFCs. The energy performance was observed at different temperatures. A variation in energy performance was observed at different temperatures. The present study was optimized from the 15 °C to 35 °C range, as shown in Figure 11b. The results showed that 25 °C offered a voltage of 21 mV for GO and 31 mV for GO-PANI, while performance at the temperature of 20 °C or 30 °C showed less energy efficiency as compared to 25 °C. The performance at a temperature of 35 °C also showed voltages of 10 mV and 12 mV for GO and GO-PANI, respectively. The energy performance varied at different temperatures, which can be interpreted in the way that temperature can disturb the biofilm and bacterial activities. The present study is more prominent and encouraging because the high-quality electrode and natural potato waste as an organic substrate empowered the bacteria to respirate, which showed some results. This means that the BMFCs can work below 20 °C with an efficient organic substrate. Similarly, at 25 °C the GO anode showed a remediation efficiency of 15% and 14% for Cd and Pb, respectively. However, the performance observed below 20 °C showed a remediation efficiency of 3 (Cd) and 2.5% (Pb), which is quite low, but this feature urged the improvement of the BMFCs to work under an unstable environment. The increment in temperature during the BMFCs operation disturbed the growth of biofilm and its activities. Mizan et al. [70] clearly stated in their work that the formation and activities of biofilm were declined at temperatures over 25 °C or below 20 °C. They preferred room temperature for the healthy growth of biofilm and for better efficiency. Previously, Li et al. [71] also studied the effect of temperature on microorganisms by operating different ranges of temperature. They also explained the detailed mechanism regarding the effect of temperature on biofilm from 10 °C to 55 °C. They recorded 2.64 W/m^3^ power density at 10 °C, and no power was recorded at 55 °C. They observed 6.34 W/m^3^ power density at 30 °C. In conclusion, they mentioned that room temperature or slightly higher, such as 24 °C to 30 °C, is better for exoelectrogens’ survival in the MFCs. In the present study, it has been observed that room temperature offered better removal efficiency as well as energy output as compared to other ranges of temperature. This can be observed in the case of GO-PANI, which also showed the highest remediation efficiency of Cd (20%) and Pb (17%) at 25 °C. The composite electrode showed quite high remediation and energy performance compared to unmodified GO, which might be due to the introduction of PANI. This means that PANI can increase the biocompatibility and electron transportation. All temperature range readings were taken after a time interval of every 10 days.

#### 3.3.3. Effect of Organic Substrate on Energy Generation and Remediation Efficiency of Cd (II) and Pb (II)

**Figure 12:**
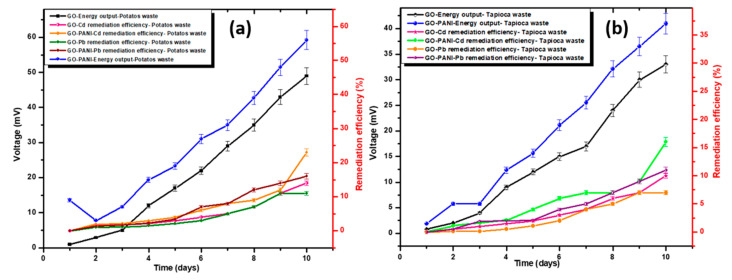
Effect of (**a**) potato waste and (**b**) tapioca waste as organic substrates on energy generation and remediation efficiency via BMFCs.

The organic substrate is one of the essential parameters to be optimized for better performance of BMFCs. In BMFCs, a natural organic substrate was used, which makes it different from others. Several researchers used different natural wastes as an organic substrate and reported their novel achievements [72,73]. Recently, in 2020, Din et al. [74] studied potato waste as an organic substrate in MFCs. They concluded that 1.12 V was the energy output, which is quite a bit higher than other organic substrates such as glucose, acetate, etc. They used sweet potato pulp in MFCs and provided the pulp every day to the bacteria. As in the present study, all the waste was supplied together in an anode chamber and sealed without providing any external supply of substrate. The maximum achieved result of the present study was 87.71 mA/m^2^ (current density) at 150Ω external resistance in 25 days of operation. Any carbohydrate natural waste can offer energy to bacteria for their respiration process, which follows the oxidation mechanism [75]. According to previous studies, there are very rare efforts present on the utilization of natural waste as an organic substrate in BMFCs. A few studies showed that cellulose vegetable waste, biomass waste, brewery wastewater, chocolate industry wastewater, mangrove waste, etc. were used as organic substrates. Additionally, in another study, Salvin et al. [76] used mangrove waste as an organic substrate in BMFCs to enhance the energy efficiency. However, to validate the performance of potato waste in the present study, another local carbohydrate-derived waste known as tapioca was used for comparative study. An equal amount (100 g) of each waste (potato and tapioca waste) was taken for optimization purposes. The results depicted in Figure 12a,b show that the sweet potato waste showed two times higher energy generation efficiency than tapioca waste. Moreover, both organic substrates are carbohydrate-based with fiber, starch, and simple sugar, but the tapioca was found to have more starch compared to the sweet potato waste. Grace et al. [77] stated that tapioca contains 88.80 ± 0.13% starch content, which is quite a bit higher than sweet potato waste (84.02 ± 1.04%). Starch is a complex carbohydrate which is not easy to digest at large amounts. Therefore, potato waste showed higher performance due to the presence of lower amounts of complex carbohydrates. The remediation efficiency of Cd (II) was 14% (GO) and 23% (GO-PANI), and for Pb (II) it was 11% (GO) and 16% (GO-PANI) in the case of sweet potato waste. In contrast, in the case of tapioca the remediation efficiency of the Cd (II) was 10% (GO) and 16% (GO-PANI), and for Pb (II) it was 7% (GO) and 11% (GO-PANI). Overall, the sweet potato waste showed more efficiency than tapioca.

## 4. Conclusions and Future Perspectives

The present study highlights the BMFCs approach to remediate the highly toxic metal ions from synthetic wastewater and simultaneously generate energy. The present study successfully showed the synthesis of the cellulosic derived modified graphene/PANI anode for improving the electron transportation, which led to high energy performance. BMFCs are one of the updated configurations of MFCs which are more eco-friendly, cost-effective, and an easily controllable technique. In BMFCs, natural waste is used as an organic substrate and there is no need to provide them from an external source. Until now, few studies were reported on the utilization of natural organic waste [78]. In the present study, sweet potato waste was used as an organic substrate with novel self-fabricated anodes to achieve the high performance of BMFCs. The prepared anodes were electrochemically supported by considering different approaches to validate the performance of energy generation and wastewater treatment. Different material characterizations and BMFCs operational results indicated that both self-fabricated electrodes are highly stable (thermal and chemical) as well as biocompatible for BMFCs operation. The healthy biofilm growth around the anodes and bacterial activities proved that the GO and GO-PANI based materials are not toxic and are also chemically stable for long-term—up to 40 days. Furthermore, two highly toxic metals (Cd (II) and Pb (II)) were operated and successfully achieved significant results in a very short period of operation. In the present study, the BMFCs were operated by using two electrodes, GO and GO-PANI, with sweet potato as an organic substrate. Each BMFCs operation took 40 days and showed highest current efficiency within 25 days. On the 25th day, the GO showed 21.92 mA/m^2^ and GO-PANI showed 87.71 mA/m^2^. Similarly, the achieved remediation efficiency of GO was 55% Cd (II) and 53.50% Pb (II), while the GO-PANI showed a remediation of 65.51% Cd (II) and 60.33% Pb (II). According to the scientific literature, there has been no work reporting on the removal of Cd (II) and Pb (II) from synthetic wastewater through BMFCs before. The concluding remarks of the present study are that the fabricated novel electrodes performed well to enhance the electron transfer rate, but the used organic waste showed less stability in BMFCs. The lower stability of the organic substrate might have affected the remediation efficiency and energy generation because the organic substrate failed to provide enough power to the bacterial community, which led to poor performance of BMFCs operation. Future studies should focus on the long-term stability of organic substrates in BMFCs for industrial practice. Several other carbohydrate-based waste materials are easily available, such as sugar cane waste, which could also be tested in BMFCs. Additionally, multiple parameter optimization was carried out by using the fabricated anode and synthetic metal ion-based wastewater, and it was concluded that a stable natural environment is preferable for BMFCs operation.

## Figures and Tables

**Table 1:** Summary of physicochemical analysis of wastewater and synthetic wastewater.

Physicochemical Properties	Raw Wastewater	Synthetic Wastewater
pH	7.07	6.45
Odor	unfriendly smell	unfriendly smell
Temperature	24–27 °C	25–27 °C
Color	Light yellow	Light yellow
Electrical conductivity	60 μS/cm	75 μS/cm
Cd (II)	0 ppm	100 ppm
Pb (II)	0 ppm	100 ppm

**Table 2:** A comparative summary of the anode electrodes in benthic microbial fuel cells (BMFCs).

Anode Electrode	Cathode Electrode	Reactor Configuration	Inoculation Source	Surface Area	Size	Power Density	Current Density Efficiency	References
Carbon fiber	Carbon fiber brush	BMFCs	*Acidobacteria* and *Actinobacteria* species	0.628 m^2^	4 × 2.5 cm	8.24 mW/m^2^	-	[46]
Graphite activated carbon	Carbon brushes	BMFCs	Mix culture	3.14 m^2^	5 × 5 cm	-	25 mA/m^2^	[47]
Titanium wires	Titanium wires	BMFCs	Real wastewater	0.756 m^2^	4 × 2 cm	35 mW/ m^2^	-	[48]
Inverted tube granular activated charcoal	carbon cloth roll	BMFCs	Mix sludge	0.25 m^2^	-	2550 mW /m^2^	-	[49]
Carbon paper	*-*	BMFCs	Lake Furnas sediment	2.2 m^2^	-	1 mW/m^2^	-	[50]
Carbon cloth	Carbon cloth	BMFCs	Mix culture bacterial community	12.56 m^2^	10 × 10 cm	-	81 mA/m^2^	[51]
GO	Graphite rod	BMFCs	Synthetic wastewater	0.0071 m^2^	7.5 × 1.2 cm	0.11 mW/m^2^	24.56 mA/m^2^	Present work
GO-PANI	Graphite rod	BMFCs	Synthetic wastewater	0.0071 m^2^	7.5 × 1.2 cm	1.1 mW/m^2^	87.71 mA/m^2^	Present work

**Table 3:** Bioremediating efficiency of prepared anodes for metal ions from synthetic wastewater via BMFCs energized by local sweet potato waste.

Electrodes		Target Metal	Inoculation Source	Initial Concentration (ppm)	Operational Time (Days)	Remediation Efficiency (%)	MCL (ppm)	
Anode	Cathode						MMOH	WHO
GO	Graphite rod	Cd (II)	Synthetic wastewater	100	0	0.00	1	0.05
					10	14.01		
					20	23.09		
					30	44.91		
					40	55.00		
GO-PANI	Graphite rod				0	0.00		
					10	22.99		
					20	36.88		
					30	52.02		
					40	65.51		
GO	Graphite rod	Pb (II)	Synthetic wastewater	100	0	0.00	0.01	0.05
					10	10.32		
					20	17.99		
					30	35.54		
					40	53.50		
GO-PANI	Graphite rod				0	0.00		
					10	16.01		
					20	29.09		
					30	48.69		
					40	60.33		

MCL = Maximum contaminant level; MMOH = Malaysian Ministry of Health; WHO = World Health Organization, Data received from reference [48,49].

**Table 4:** List of identified bacterial species from clone libraries of biofilm on surface of self-fabricated anode.

Accession Number (16S rRNA Gene)	Bacteria Species	Query Cover (%)	Identity (%)
**GO anode bacterial species in synthetic wastewater**			
NR_114074.1	*Acinetobacter radioresistens* strain NBRC 102413	98	97.8
NR_148248.1	*Bacillus bingmayongensis* strain FJAT-13831	92	96.5
NR_170494.1	*Bacillus fungorum* strain 17-SMS-01	92	96.4
NR_152692.1	*Bacillus wiedmannii* strain FSL W8-0169	91	96.6
NR_026210.1	*Acinetobacter radioresistens* strain FO-1	98	96.9
NR_147743.1	*Fluviicoccus keumensis* strain 4DR5	91	90.8
NR_109730.1	*Paraperlucidibaca wandonensis* strain WT-RY4	95	88
NR_114419.1	*Proteus mirabilis* strain ATCC 29906	92	94
NR_043648.1	*Xenorhabdus hominickii* strain KE01	92	92
NR_043999.1	*Cosenzaea myxofaciens* ATCC 19692	92	93.4
**GO-PANI anode bacterial species in synthetic wastewater**			
NR_028624.1	*Bacillus funiculus* strain NAF001 16S	93	98
NR_114074.1	*Acinetobacter radioresistens* strain NBRC 102413	98	97.8
NR_134685.1	*Acinetobacter variabilis* strain NIPH 2171	97	96.8
NR_113237.1	*Acinetobacter baumannii* strain JCM 6841	98	94
NR_104948.1	*Prolinoborus fasciculus* strain CIP 103579	93	94
NR_148248.1	*Bacillus bingmayongensis* strain FJAT-13831	92	96.5
NR_170494.1	*Bacillus fungorum* strain 17-SMS-01	92	96.4
NR_043997.1	*Proteus mirabilis* strain NCTC 11938	94	91.31
NR_114419.1	*Proteus mirabilis* strain ATCC 29906	92	94
NR_152692.1	*Bacillus wiedmannii* strain FSL W8-0169	91	96.6

## Data Availability

Data is contained within the article.
